# Impact assessment of fortified rice under the public distribution system: evidence from the tribal regions of Gujarat

**DOI:** 10.3389/fpubh.2026.1757408

**Published:** 2026-04-01

**Authors:** Deepa Tiwari, Sirimavo Nair

**Affiliations:** Department of Food and Nutrition, Faculty of Family and Community Sciences, The Maharaja Sayajirao University of Baroda, Vadodara, Gujarat, India

**Keywords:** folate, fortified rice, haemoglobin, targeted public distribution system, tribal, vitamin B_12_

## Abstract

**Introduction:**

Staple food fortification is a globally recognized method for reducing micronutrient deficiencies. India started its food fortification journey in the 1950’s with the fortification of Vanaspati. Over the years, several food vehicles, such as double fortified salt, oil, wheat flour, and rice, have been studied. Based on the mixed results observed, the Government of India launched the Central pilot scheme on “Fortification of rice and its distribution under the public distribution system” in 2019. This scheme was launched across 15 districts of India. The major aim of this study was to assess the impact of routine consumption of fortified rice distributed via the Targeted Public Distribution System (TPDS) on haemoglobin, ferritin, folate and vitamin B_12_ levels.

**Methods:**

A simple random sampling method was used to enroll 452 women of reproductive age. Using a purposive sampling technique and inclusion and exclusion criteria, a total of 167 WRA were enrolled for the intervention phase. Fortified rice procured from the Targeted public distribution system was monitored for a period of 6 months and 1 year. Micronutrient level was found to be as per the FSSAI, 2018 regulations (Iron, 40.13 mg; Folate, 93.66 µg; and Vitamin B_12_, 0.89 µg per kg of fortified rice).

**Results:**

It was found that fortified rice intervention helped in improving hemoglobin levels (capillary group: 0.52 g/dL; *p* = 0.005 and venous group: 0.55 g/dL; *p* = 0.001); however, no significant improvements were observed in serum ferritin, folate, and vitamin B_12._

**Conclusion:**

Routine consumption of fortified rice significantly improved haemoglobin levels; however, no significant improvements were observed in ferritin, folate, and vitamin B_12_ levels.

## 1 Introduction

Anemia is a significant public health concern worldwide (1). Studies reveal that anemia is a multifactorial concern where several genetic, environmental, and dietary factors contribute to anemia (2). Studies carried out in India shows that among all the contributory factors, iron deficiency anemia is one of the leading causes (3, 4). According to the National Family Health Survey IV and V, the prevalence of anemia among Women of reproductive age (WRA) has increased from 53% to 57%. The situation is even worse in the tribal areas of the country, where the percentage goes to more than 70% (5). Some of the key strategies recommended for the prevention of anemia are supplementation of iron and folic acid in conjunction with deworming; staple food fortification with iron, folic acid, and vitamin B_12_; dietary diversification and public health measures, such as controlling malaria, worm infestations, and other illnesses (6).

Anemia has always been in the priority list under the National Health policies of India. Though there have been several existing strategies recommended from time to time, the situation of anemia is more or less the same. According to the policy guideline of the Ministry of Health and Family Welfare, under the ambit of Anemia Mukt Bharat, the aim was to reduce the burden of anemia by 3 percentage points every year from 2018 to 2022 by implementing a 6 × 6 × 6 intervention approach. The intervention approaches include addressing the non-nutritional causes of anemia in endemic pockets, with special focus on malaria, haemoglobinopathies, and fluorosis; mandatory provision of iron–folic-acid fortified foods in public health programs; testing of anemia using digital methods and point of care treatments; intensified year-round behavior change communication; prophylactic iron and folic acid supplementation; and deworming. Among all the approaches, intervention of fortified foods was also considered to be a potential approach (7).

In view of the same, the Government of India launched the Centrally Sponsored Pilot Scheme on “Fortification of Rice and its Distribution under Public Distribution System” on 14 February 2019. The scheme was rolled out in a phased manner, where 100% coverage was achieved by March 2024. This program was started on a pilot basis across 15 districts of India. The Aspirational district of Narmada was one of the selected pilot districts under the scheme (8). Therefore, the present study was conceptualized to understand the impact of routine consumption of fortified rice distributed via the TPDS on haemoglobin, serum ferritin, folate and Vitamin B_12,_.

## 2 Methods

### 2.1 Study area, population, and sampling

**Figure 1:**
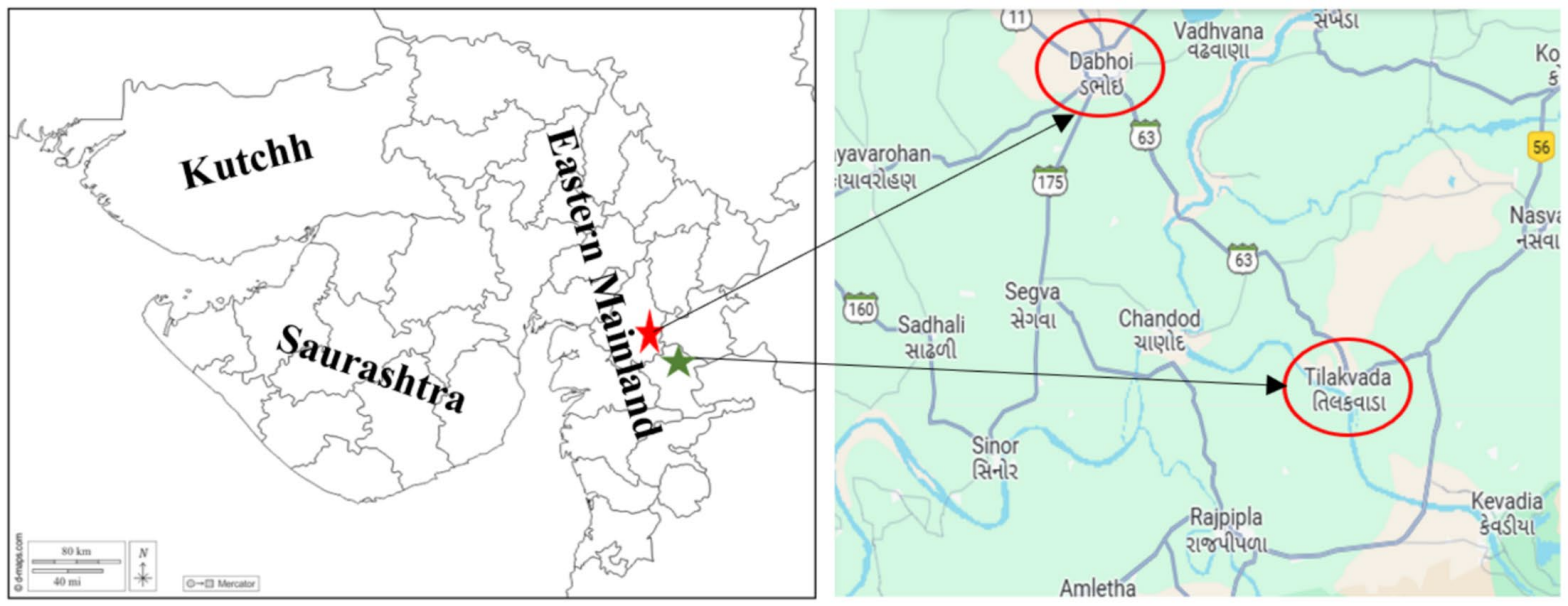
Selection of the study site.

A community-based, quasi-experimental longitudinal study embedded under the Targeted Public Distribution System was conceptualized to assess the impact of fortified rice intervention on hemoglobin and serum ferritin, folate, and vitamin B_12_. The study was carried out in the rural areas of the tribal region of Gujarat, India. Geographically, the state of Gujarat can be divided into three regions: Kutch, Saurashtra, and the Eastern mainland region. The eastern mainland region is predominantly inhabited by tribal communities. Among the eastern mainland region, the aspirational district of Narmada and Vadodara were selected for the study, as illustrated in Figure 1. Narmada district constitutes five blocks or talukas, viz. Tilakvada, Nandod, Sagbara, Garudeshwar, and Dediapada, while Vadodara district constitutes 11 blocks, viz. Savli, Vadodara-Rural, Waghodia, Dabhoi, Padra, Karjan, Shinor, Desar, Vadodara city-east, Vadodara city-south, Vadodara city-west, and Vadodara city-north. After analyzing the required logistical support, Dabhoi taluka from Vadodara district and Tilakvada taluka from the aspirational district of Narmada were purposively selected. The sample size was calculated based on the data of the National Family Health Survey-IV, 2015–16 (9). The following formula was used for calculating the sample size. Total sample, $n=(\mathrm{Z\alpha}/2)\;2p(1-q)/d^{2}$ . For 95% confidence interval, the value of Z_α_ (critical value from the standard normal distribution) is 1.96, p = prevalence of anemia among women (15–49 years of age) = 57.6%, *q* = (100−p) = 42.4%, and d = desired level of precision = ±5%. Therefore, the total sample size of the study was *n* = 375.28 ≈ 376 (using 20% attrition) = 452.

This study was carried out according to the guidelines of the Helsinki Declaration, 1964. All the subjects were briefed with the objectives of the study, and written Informed consent was obtained. Subjects were free to leave the study at any point during the study period. The Institutional Ethical Committee granted approval on 9 July 2022 (IECHR/FCSc/PhD/2022/6). This study was also registered under the Indian Council of Medical Research-Clinical Trials Registry on 11/07/2022 (CTRI/2022/07/043903).

In order to assess the availability of fortified staples in the study area, a market survey was conducted. Fortified staples such as fortified wheat flour, fortified milk, and fortified rice were only available in the Government social safety net programs, *such as* Targeted Public Distribution System (TPDS), Pradhan Mantri Poshan Shakti Nirman (PM-POSHAN), Integrated Child Development Services (ICDS) scheme, and Pradhan Mantri Garib Kalyan Anna Yojna (PMGKAY). The PMGKAY scheme was launched to bring relief due to economic disruption caused by the COVID-19 pandemic. Under the scheme, 5 kg of food grains (Wheat and rice) were given free of cost to the priority households. Under the TPDS, 3 kg of fortified rice was allocated per person per month to the Below the Poverty Line (BPL) families, while 35 kg of food grains (10 Kg rice and 15 Kg wheat) was distributed to the Antyodaya Anna Yojna (AAY) beneficiaries. In this study, beneficiaries belonging to the Priority Households (PHHs) were studied.

In India, considering the climatic factors and preferred cooking preparations, the hot extrusion method is recommended for preparing fortified rice. Fortified rice distributed under the TPDS was prepared using rice flour, Potable water (IS 10500: 2012), FSSAI-approved food grade vitamin and mineral premix, acid regulators, and emulsifiers (Penta-sodium Triphosphate – INS 451 (i), citric acid INS 330, etc., was used for preparing fortified rice kernel (FRK). According to the Food Safety and Standards Authority of India’s guideline, micronutrients such as iron, folic acid, and vitamin B_12_ should be in the following range: 28 mg–42.5 mg of iron, 75 μg–125 μg of folic acid, and 0.75 μg–1.25 μg of vitamin B_12_ per Kg of fortified rice, respectively. A fortified rice kernel or FRK is a reconstituted rice grain made up of rice flour and a premix mixture. When the FRK is mixed with the regular rice in a ratio of 1:100, it is known as fortified rice (10).

In this study, fortified rice distributed from the TPDS platform was used for assessing the impact of the intervention. Simple random sampling was used for enrolling the women of reproductive age for the baseline phase; however, the purposive sampling method was employed to enroll WRA for the intervention phase. The following inclusion criteria were followed: subjects having BPL/AAY ration cards and procured fortified rice from the fair price shops, individuals residing in Narmada and Vadodara districts of Gujarat, subjects consented for the study, mild or moderate anemic subjects, and adult women aged between 15 and 45 years. In the following cases subjects were excluded from the study *viz*. subjects did not procure fortified rice or procured but thrown Fortified rice kernels, Individuals resided in blocks other than Tilakvada and Dabhoi taluka, Individuals with chronic illnesses such as Tuberculosis, Diabetes, and other non-communicable diseases, Individuals on some kind of medication, and subjects with sickle Cell anemia or Thalassemia.

**Figure 2:**
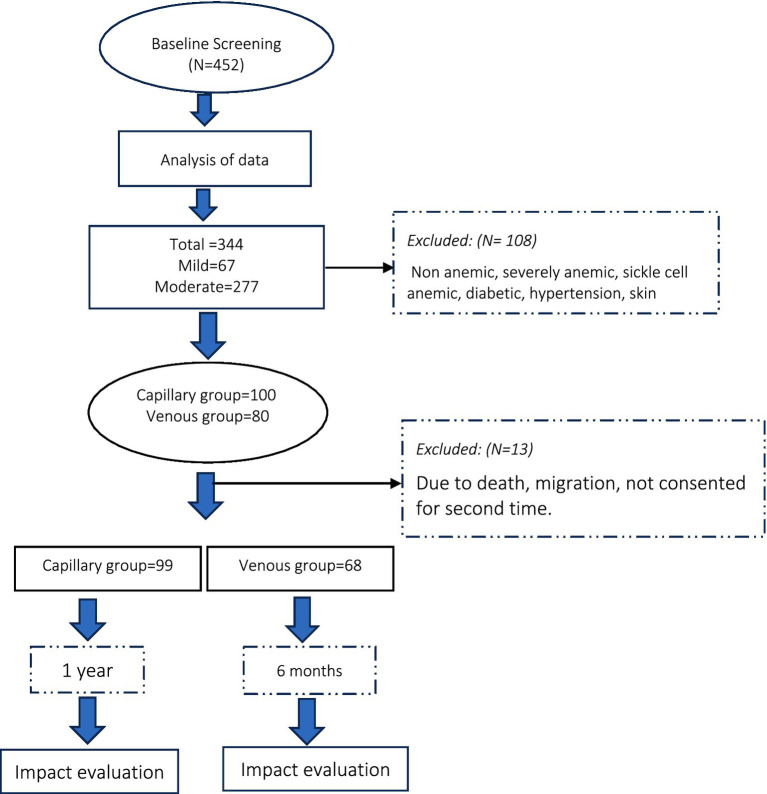
Screening of the subjects.

As illustrated in Figure 2, this study conducted baseline screening of 452 subjects. The analysis showed that approximately 61.28% (277) were moderately anemic, followed by 14.82% (67) mildly anemic, 7.30% (33) severely anemic, and 16.59% (75) non-anemic. Approximately 108 (23.89%) severe and non-anemic subjects were excluded due to not fulfilling the inclusion criteria. Out of 344 WRA, approximately 180 subjects consented for the study. Among 180 intervention subjects, 100 consented to capillary blood samples, while only 80 consented to venous blood samples. Subjects in the capillary group (CG) were monitored for a period of 1 year, while subjects in the venous group (VG) were monitored for a period of 6 months. Out of 180 intervention subjects, approximately 167 subjects completed the study. All those subjects who procured fortified rice and did not throw the fortified rice kernels (FRK) were enrolled under the experimental group, while those who did not consume FRK or those who consumed only farm-grown rice were enrolled under the control group.

### 2.2 Survey instrument

A pretested and structured questionnaire was developed for recording the responses. The questionnaire was administered in the local (Gujarati) language during daytime hours. The following components were included in the questionnaire: Kuppuswammy socioeconomic scale, 2021 (11); height, weight, mid-upper arm circumference (12), and hemoglobin (13); and 24-h dietary recall questionnaire (14).

### 2.3 Anthropometric and biochemical assessment

Anthropometric measurements, such as height, were measured to the nearest 0.1 cm using a stadiometer. Subjects were asked to stand erect, and it was ensured that shoulder blades, buttocks, and heels touch the measurement rod, while the head touches the Frankfurt plane. The weight of the subjects was measured using a digital weighing scale (Omron HN 289 Weighing Scale). Mid-upper arm circumference (MUAC) assessment was carried out using a flexible, non-stretched tape made of fiberglass. All the subjects were asked to stand erect, sideways to the measurer, with the head on a Frankfurt plane and with a relaxed arm. Measurement was taken at the midpoint of the upper-left arm between the acromion and the tip of the olecranon process (10). After locating the mid-point of the upper left arm, MUAC tape was wrapped gently around the arm, and measurements were taken to the nearest millimeter. Height for age *Z*-score (HAZ) and body mass index-for-age *Z*-score (BAZ) were calculated using National Center for Health Statistics (NCHS)/ World Health Organization (WHO) references (15).

Blood samples were collected by a trained phlebotomist. A total of 5 mL of venous blood samples were drawn into a standard EDTA red-top vacutainer tube. All EDTA tubes were wrapped in aluminum foil to protect from sunlight, stored in a dry ice box, and transported to the testing laboratory, where samples were kept at −80 °C until analysis. On the day of analysis, the temperature of samples, calibrators, and controls was checked to ensure it was maintained between 20 °C and 25 °C. Hemoglobin analysis was carried out within 24 h using a three-part automated hematology analyzer (Nihon-Kohden, Celltac Alpha MEK-6420P, Japan). Ferritin, folate, and vitamin B_12_ were analyzed using the SIEMENS ADVIA Centaur and ADVIA Centaur XP immunoassay system. Sensitivity and assay range of ferritin, folate, and vitamin B_12_ were 0.5–1,650 ng/mL (1–3,630 pmol/L), 0.35–24 ng/mL (0.79–54.36 nmol/L), and 45–2000 pg./mL (33–1,476 pmol/L), respectively (16). Capillary blood samples were analyzed using Drabkin’s method. Exactly 20 μL of blood sample were drawn from the subjects using capillary tubes. Blood-filled capillaries were poured over the Whatman No. 2 filter paper, and blood spots were dried and stored in the airtight polyethylene zip bags. Blood-filled dry filter paper was placed overnight in 5 mL of Drabkin reagent. The absorbance of the sample and the standard was measured against a reagent at 540 nm in a Systronics spectrophotometer-117 (13). Samples of fortified rice were analyzed in two National Accreditation Board for Testing and Calibration Laboratories (NABL), Nagpur and Anand. Analysis of iron, folate, and vitamin B12 was conducted according to the standardized methods prescribed by the FSSAI, viz. FSSAI.FR.16.001.2022, FSSAI.FR.16.002.2022, and FSSAI.FR.16.003.2022 (17).

### 2.4 Statistical analyses

The collected data were subjected to statistical analyses. Descriptive statistics were used to analyze the distribution of the variables. Dropouts were excluded from the analysis. Anthropometric data were presented in mean ± standard deviation (SD) terms. Dietary data of the subjects was found to be non-normal, therefore, data were presented in both mean ± standard deviation (SD) and median and inter quartile range (IQR). Chi-squared test of independence was used to assess categorical variables, such as socioeconomic parameters. Analysis of covariance (ANCOVA) was used to assess the impact of fortified rice intervention on the micronutrient level. Analyses were performed on R statistical software (18).

## 3 Results

### 3.1 Availability of fortified Staples in the study area

All the subjects procured raw ration such as wheat, rice, iodized salt, pulses (tuver dal and channa dal), and sugar from the fair price shops. All the subjects availed the subsidized food commodities as per the following rates: wheat (₹2), fortified rice (₹3), sugar (₹22), iodized salt (₹1), tuver dal (₹50), channa dal (₹30), and edible oil (₹100) per kg/liter. Edible oil was mostly given during the Diwali and Janmashtami celebrations. Fortified wheat flour was only available under the integrated child development services (ICDS) scheme. All the Anganwadi workers used fortified wheat flour for preparing hot cooked meal (HCM) for children below 6 years. Double fortified salt was discontinued in the year 2022 due to the overlap of salt distribution between the Department of Food, Civil Supplies and Consumer Affairs and the Department of Women and Child Development. Therefore, only iodized salt was available for the general population’s consumption. None of the households were found to be consuming loose salt. Doodh Sanjeevani Yojna (DSY) was also operational in the study area, in which 100 mL of milk was provided 5 days a week to children below 6 years, and 200 mL of milk was given to primigravida two times a week. Fortified rice was available under (PM-POSHAN), TPDS, PMGKAY, and ICDS scheme. In a nutshell, only fortified rice (fortified with folate, iron, and vitamin B_12_), and edible oil (fortified with vitamin A and D) were available for the general population consumption.

### 3.2 Background information

Households were classified into different socioeconomic categories using the Kuppuswammy socioeconomic scale, 2021. The classification was based on three variables such as occupation of the household head, education of the household head, and per capita income of the household. Table 1 illustrates a detailed description of the socioeconomic conditions of the subjects. The majority of the women were educated up to secondary level. Agriculture and animal husbandry were the major occupational activities among women. Joint family structure was found to be more prevalent in the study area. Semi-pacca settlements (walls made up of brick, cement/stone, and mud with a tin roof) were found to be more prevalent. The majority of the subjects were below the poverty line (BPL) and had Antyodaya Anna Yojna (AAY) ration cards. Subjects were classified into different classes, out of which the majority of the women, that is, 84.73% belonged to the upper lower class, followed by lower-middle (8.84%), and lower class (6.41%), respectively.

**Table 1:** Socioeconomic status of the subjects.

Parameters	Categories	Number (frequency)
Age of the respondents	16–19 years	119 (26.32)
	20–30 years	92 (20.35)
	31–40 years	151 (33.40)
	41–45 years	90 (19.91)
Education of the respondents	Primary	18 (3.98)
	Secondary	356 (78.76)
	Higher Secondary	38 (8.40)
	Graduation	6 (1.32)
	Others	34 (7.52)
Occupation of the respondents	Daily wage	120 (26.54)
	Farming	319 (70.57)
	Salaried	13 (2.87)
Type of family	Joint	377 (83.40)
	Nuclear	75 (16.59)
Number of family members	<5	71 (15.70)
	<10	364 (80.53)
	>10	17 (3.76)
Type of house	Kacha house	5 (1.10)
	Semi pacca house	236 (52.21)
	Pakka pacca house	211 (46.68)
Ration card	BPL	421 (93.14)
	AAY	31 (6.85)
SES status	Lower	29 (6.41)
	UL	383 (84.73)
	LM	40 (8.84)

A total of 452 women of reproductive age participated in the study, out of which 332 were aged between 20 and 45 years, while 120 were aged between 16 and 19. Analysis of the anthropometric measurements of the subjects revealed that approximately 43.37% subjects had normal BMI, followed by chronically energy deficient (24.39%), obese (22.59%), and overweight (9.63%). Mean BMI among adult women (*n* = 332) was 21.6 ± 4.26 kg/m^2^. The mean MUAC score of the women of reproductive age was 25.4 ± 3.49 cm. Mean height-for-age Z-score (HAZ) among adolescent girls (*n* = 120) was −1.22 (0.85). Approximately 15% adolescent subjects had HAZ ≤ 2 SD, followed by HAZ ≤ 3 SD (2.5%). Mean BMI-for-age Z-score (BAZ) was −1.09 (1.07). Approximately, 18.3% had BAZ ≤ 2 SD, followed by BAZ ± 1SD (5%), BAZ ≤ 3 SD (2.5%), and BAZ ± 2 SD (0.83%), respectively. Mean MUAC score among adolescents was 22.92 ± 2.95 cm. Mean hemoglobin level among WRA (*n* = 452) was 10.2 ± 1.59 (5.2–14.8) g/dl. Approximately, 61.28% were moderately anemic, followed by mildly anemic (15.2%), severely anemic (7.3%), and non-anemic (16.59%), respectively.

BMI data of the capillary group subjects was found to be non-normal (*p* = 0.007); therefore, Mann–Whitney-U test was performed. It was observed that the median BMI of the subjects of the control group (*n* = 44) was 21.3 (7.49) kg/m^2^, while in the experimental group (*n* = 55), it was 20 (4.2) kg/m^2^ at *p* = 0.40. Mean MUAC of the control and experimental subjects was 24.9 ± 4.12 cm and 25.2 ± 3.41 cm at *p* = 0.70, respectively. At the baseline, approximately 48 subjects were moderately anemic, while 7 subjects were mildly anemic in the experimental group. In the control group, approximately 35 subjects were moderately anemic, while 9 subjects were mildly anemic. Among venous groups, the median BMI of the subjects of the control group was 21.6 (7.42) kg/m^2,^ and 21.3 (6.81) kg/m^2^ for the experimental group at *p* = 0.71. Mean MUAC score of CG was 25.6 ± 3.23 cm and 25.2 (3.63) cm among the EG at *p* = 0.68. In the control group, 21 subjects were mildly anemic, followed by moderately anemic (13), while in the experimental group, approximately 14 subjects were moderately anemic, followed by mildly anemic (19).

### 3.3 Nutrient intake of the subjects

Table 2 illustrates that the mean intake of macro- and micronutrients was found to be similar across the study area, except for thiamine, riboflavin, and phosphorus. Table 3 demonstrates that compared with the recommended dietary allowances (RDA), the current dietary pattern of the subjects met only 50%–75% of the recommended energy requirement (19). The mean carbohydrate and fat intake were found to be higher than the recommended levels, while the majority of subjects consumed only 50%–75% of the recommended protein intake. In comparison to macronutrients, the intake of micronutrients, such as iron, folate, ascorbic acid, thiamine, riboflavin, niacin, and calcium, was found to be significantly lower than the recommended levels. Notably, despite rearing milch animals, calcium intake remained very low. One of the contributing factors of low calcium intake was predominant consumption of milk in the form of tea, coffee, and buttermilk, rather than as a standalone beverage. Furthermore, only a limited proportion of respondents reported daily milk consumption. A major portion of the milk was sold to the nearby dairies.

**Table 2:** Mean nutrient intake among WRA (*N* = 167).

Nutrients	Group	Mean	SD	SE	95% CI		Median	IQR	Minimum	Maximum	*p*-value
					Lower	Upper					
Energy	Control	1849	160.57	18.07	1813	1885	1847	187	1,524	2,369	0.05
	Experimental	1792	209.26	22.31	1748	1837	1850	262	1,186	2,240	
Protein	Control	36.42	5.64	0.63	35.16	37.68	37.52	7.39	19.62	49.33	0.13
	Experimental	34.98	6.71	0.72	33.56	36.40	34.58	11.63	24.03	52.74	
Fat	Control	69.24	11.11	1.25	66.75	71.73	68.73	13.56	46.25	103.23	0.29
	Experimental	67.55	9.38	1.00	65.56	69.54	66.22	13.82	46.97	95.14	
Carbohydrate	Control	270.70	28.93	3.25	264.22	277.18	278.57	32.84	184.60	334.71	0.83
	Experimental	269.84	22.76	2.43	265.01	274.66	270.78	33.70	205.84	322.24	
Thiamine	Control	0.85	0.28	0.03	0.79	0.91	0.85	0.21	0.37	1.94	0.01
	Experimental	0.76	0.21	0.02	0.71	0.80	0.74	0.28	0.36	1.64	
Riboflavin	Control	0.51	0.15	0.02	0.47	0.54	0.49	0.17	0.25	1.00	0.04
	Experimental	0.46	0.14	0.01	0.44	0.49	0.43	0.20	0.25	0.99	
Niacin	Control	6.50	1.44	0.16	6.18	6.83	6.43	1.98	3.73	11.35	0.37
	Experimental	6.28	1.80	0.19	5.90	6.66	6.13	2.69	2.99	12.54	
Folates	Control	163.00	32.52	3.66	155.71	170.28	161.11	36.09	87.00	308.01	0.19
	Experimental	155.41	41.20	4.39	146.68	164.14	152.07	48.27	59.01	283.35	
Ascorbic acid	Control	52.37	22.78	2.56	47.27	57.47	46.45	33.88	18.20	114.31	0.40
	Experimental	49.54	20.83	2.22	45.12	53.95	44.26	21.76	19.45	121.20	
Calcium	Control	231.22	65.30	7.35	216.59	245.85	214.06	83.49	120.46	444.44	0.90
	Experimental	232.40	63.23	6.74	219.00	245.80	239.64	91.15	98.57	414.70	
Iron	Control	11.8	2.40	0.27	11.3	12.4	11.4	3.20	7.20	18.45	0.09
	Experimental	11.2	2.16	0.23	10.8	11.7	10.5	2.10	8.27	17.90	
Phosphorous	Control	714.68	150.96	16.98	680.87	748.49	747.27	197.81	367.17	1016.41	0.04
	Experimental	664.08	166.73	17.77	628.75	699.41	659.22	217.64	242.46	1124.36	

Note: Data were found to be normal; therefore, independent sample *t*-test was performed. Control group (Capillary group = 44, venous group = 34; Total = 78) and Experimental group (Capillary group = 55, venous group = 34; Total = 89).

**Table 3:** Nutrient consumption as per the recommended RDA.

Nutrients	Age group	RDA	50%	50%–75%	>75%	100%
*Energy (Kcal)	Adolescent girls	2,500	0.54	81.08	13.51	—
	Adult women	2,130	—	4.61	95.38	—
Carbohydrate (g)	Adolescent girls	45%–50% of TE	—	—	—	100%
	Adult women	45%–50% of TE	—	—	—	100%
Protein (g)	Adolescent girls	46	—	56.75	43.24	—
	Adult women	46	0.76	40	59.23	—
Fat (g)	Adolescent girls	35	—	—	—	100%
	Adult women	25	—	—	—	100%
Iron (mg)	Adolescent girls	32	94.59	0.54	—	—
	Adult women	29	89.23	10.76	—	—
Calcium (mg)	Adolescent girls	1,050	100	—	—	—
	Adult women	1,000	100	—	—	—
Folates (μg)	Adolescent girls	270	48.64	51.35	—	—
	Adult women	220	3.84	56.15	40	—
Ascorbic acid (mg)	Adolescent girls	70	35.13	45.94	18.91	—
	Adult women	65	12.30	33.84	53.84	—

Note: Energy values are expressed in estimated average requirement (EAR); TE: Total Energy.

### 3.4 Impact of fortified rice on hemoglobin level

Age distribution of the intervention subjects was found to be normal at *p* = 0.14. The average age among the control group (*n* = 44) was 34.93 ± 9.13 years, followed by 29.72 ± 9.40 years in the experimental group (*n* = 55). Median weight of the control group was 51.5 (16.9) kg, while in the experimental group, it was 45 (14.2) kg at *p* = 0.01. The weight of the subjects was improved to 54.1 (15) Kg and 46.5 (15) Kg, respectively, in the control and experimental group at *p* = 0.01. In both groups, weight was increased significantly compared to the baseline values. Blood samples of the capillary group were drawn at the baseline and endline (1-year) interval. Hemoglobin levels among subjects in both the experimental and control groups were assessed for normality. The distribution of the hemoglobin data was found to be normal in the experimental group (*p* = 0.77) and control group (*p* = 0.18). Table 4 shows that in the experimental group, the mean hemoglobin level at the baseline was 10 ± 0.85 g/dL, which slightly decreased to 9.85 ± 1.20 g/dL at the endline. The mean difference of 0.15 g/dL was not found to be statistically significant (*p* = 0.25), which indicated that hemoglobin level remained largely stable over the study period. In contrast, the control group showed a marked reduction from 10 ± 0.94 g/dL to 9.33 ± 0.89 g/dL (mean difference = 0.65 g/dL; *p* < 0.001).

**Table 4:** Comparison of pre- and post-haemoglobin values of control and experimental subjects—Capillary group.

	Group	Mean±SD	Median (IQR)	SE	95% Confidence interval		Min	Max
					Lower	Upper		
Hb (0 month)	Control	10.01 ± 0.94	10 (1.35)	0.14	9.72	10.30	8.00	11.6
	Experimental	10.01 ± 0.85	10 (1.25)	0.11	9.77	10.24	8.00	11.9
Hb (12 months)	Control	9.33 ± 0.89	9.15 (1.25)	0.13	9.06	9.60	8.10	12.6
	Experimental	9.85 ± 1.20	9.9 (1.65)	0.16	9.53	10.18	8.00	13.3

Note: Control group (*n* = 44) and Experimental group (*n* = 55).

Analyzing the routine consumption of fortified rice intervention on hemoglobin level, ANCOVA was performed.

**Figure 3:**
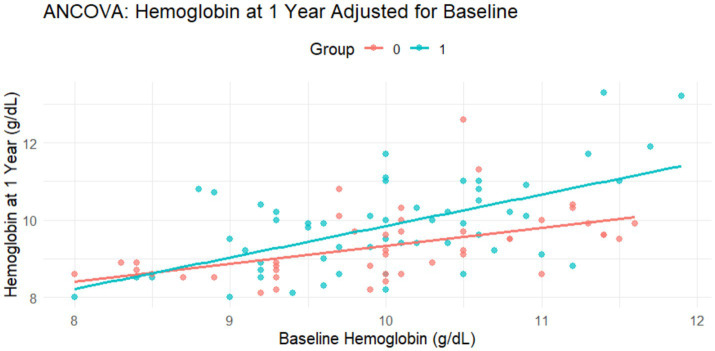
Impact assessment of fortified rice at 1 year of intervention of capillary group: (0, control group and 1, experimental group).

Figure 3 illustrates that the model was found to be statistically significant, *F* (2, 96) = 23.30, *p* < 0.001, and showed that the combination of baseline hemoglobin and group assignment explained a meaningful proportion of the variance in hemoglobin levels at 1 year. The model accounted for approximately 31.3% of the variance in endline hemoglobin levels (Adjusted *R*^2^ = 0.313). Baseline hemoglobin level was also found to be a significant covariate (*β* = 0.64, *p* < 0.001), suggesting a strong positive association between initial and follow-up values. After adjusting for baseline levels, subjects in the intervention group had significantly higher hemoglobin levels at 1 year compared to those in the control group (*β* = 0.52, *p* = 0.005). On an average, the intervention group demonstrated an increase of 0.52 g/dL in hemoglobin relative to the control group. These findings suggested that the impact of fortified rice distributed via the targeted public distribution system had a statistically significant and sustained positive effect on hemoglobin level over a 1-year period.

**Figure 4:**
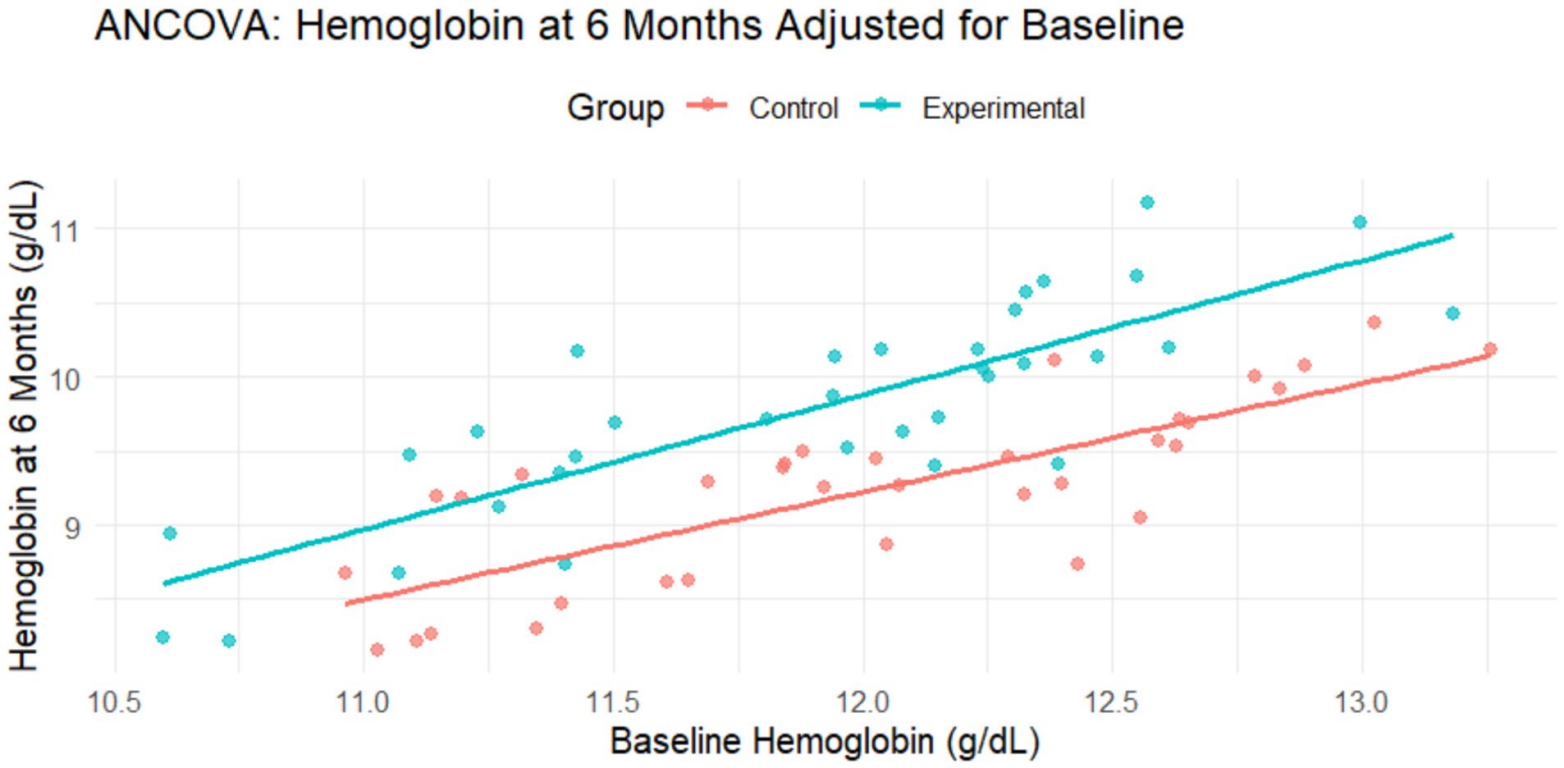
Impact assessment of fortified rice at 6 months of intervention (venous group).

Among the venous group, all the intervention subjects were mildly and moderately anemic. In the experimental group, the average age was 33.43 ± 17.97 years, while in the control group, the average age was 26.51 ± 11.10 years. Table 5 illustrates that the hemoglobin level in the control group was reduced by 0.35 g/dL (10.90 ± 0.86–10.55 ± 0.86 g/dL), while in the experimental group, it increased by 0.21 g/dL (10.84 ± 0.81–11.05 ± 1.02 g/dL). The difference was found to be significant at *p* = 0.001. In order to assess the impact of the intervention of fortified rice, analysis of co-variance (ANCOVA) was performed. Figure 4 illustrates that the model revealed a statistically significant effect of the intervention [*F* (2,65) = 34.58, *p* < 0.001], with the experimental group showing a greater increase in hemoglobin compared to the control group. After adjusting for baseline values, the experimental group had, on an average, 0.55 g/dL higher hemoglobin at 6 months compared to the control group (*β* = 0.54, *p* = 0.001). Baseline hemoglobin was also found to be a significant covariate (*β* = 0.78, *p* < 0.001), indicating that initial hemoglobin levels were strongly associated with hemoglobin at follow-up. The model accounted for approximately 51.6% of the variance in endline hemoglobin levels (adjusted *R*^2^ = 0.50).

**Table 5:** Comparison of pre and post values of subjects—venous group.

	Group	Mean ± SD	SE	95% Confidence interval		Median (IQR)	Min	Max	*p*-value
				Lower	Upper				
Hb (Pre)	Control	10.90 ± 0.86	0.14	10.59	11.20	11.10 (0.75)	8.40	11.8	0.75
	Experimental	10.84 ± 0.81	0.13	10.56	11.13	11.25 (0.80)	8.90	11.8	
Hb (Post)	Control	10.55 ± 0.86	0.14	10.25	10.85	10.90 (0.90)	8.00	11.9	0.01
	Experimental	11.05 ± 1.02	0.17	10.69	11.41	11.30 (1.27)	8.50	14.0	
Ferritin (pre)	Control	39.01 ± 33.33	5.71	27.38	50.64	29.34 (34.40)	3.00	132.6	0.63
	Experimental	30.62 ± 20.45	3.50	23.48	37.75	29.30 (24.48)	3.90	97.5	
Ferritin (post)	Control	33.85 ± 25.68	4.40	24.89	42.81	26.65 (31.97)	3.50	121.0	0.01
	Experimental	20.86 ± 17.61	3.02	14.71	27.00	16.95 (14.92)	3.50	97.9	
Folate (pre)	Control	6.98 ± 3.95	0.67	5.60	8.36	5.20 (4.65)	2.60	17.4	0.002
	Experimental	9.44 ± 3.53	0.60	8.20	10.67	8.45 (4.65)	4.20	20.0	
Folate (post)	Control	7.17 ± 3.94	0.67	5.80	8.55	5.70 (4.96)	3.20	19.1	0.008
	Experimental	9.20 ± 3.58	0.61	7.95	10.45	8.50 (4.65)	3.60	20.0	
B_12_ (pre)	Control	431.15 ± 252.84	43.36	342.93	519.37	390.0 (307.25)	148	991	0.36
	Experimental	522.44 ± 300.12	51.47	417.72	627.16	530.0 (619.25)	148	911	
B_12_ (post)	Control	256.0 ± 118.07	20.24	214.80	297.20	205.50 (143.50)	106	546	0.58
	Experimental	226.53 ± 79.39	13.61	198.83	254.23	202.50 (62.0)	105	441	

Note: Control group (*N* = 34); Experimental group (*n* = 34).

### 3.5 Impact on serum Ferrin, folate & vitamin B_12_

Ferritin level in the control group subjects was decreased from 39.01 ± 33.33 μg/L to 33.85 ± 25.68 μg/L (*p* < 0.001), while in the experimental group, it was also reduced from 30.62 ± 20.45 μg/L to 20.86 ± 17.61 μg/L (*p* < 0.001). An analysis of covariance (ANCOVA) was conducted to assess the effect of the fortified rice intervention on serum ferritin level at 6 months, controlling for baseline ferritin levels. The model was found to be statistically significant, *F*(2,65) = 44.95, *p* < 0.001, and accounted for approximately 58% of the variance in ferritin at endline (adjusted *R*^2^ = 0.567). Baseline ferritin was a strong positive predictor of ferritin at 6 months (*β* = 0.59, *p* < 0.001), indicating that higher initial ferritin levels were associated with higher levels at follow-up. However, after adjusting for baseline levels, the experimental group had significantly lower ferritin level compared to the control group (*β* = −8.07, *p* = 0.032). This suggested that the fortified rice intervention over the 6-month period did not improve ferritin levels among WRA.

Folate level among the control group subjects was changed from 6.98 ± 3.95 μg/L to 7.17 ± 3.94 μg/L (*p* = 0.5), while in the experimental group, it was changed from 9.44 ± 3.53 μg/L to 9.20 ± 3.58 μg/L, (*p* = 0.01). An analysis of covariance was conducted to determine the effect of the fortified rice intervention on serum folate levels at 6 months, while adjusting for baseline folate concentrations. The overall model was statistically significant *F*(2, 65) = 24.99, *p* < 0.001, and explained approximately 43.5% of the variance in endline folate concentrations (adjusted *R*^2^ = 0.417). Baseline folate levels were found to be a strong and significant predictor of folate at 6 months (*β* = 0.63, *p* < 0.001), indicating that individuals with higher initial levels tended to maintain higher levels at post-intervention. However, the effect of the intervention was not found to be statistically significant. After controlling for baseline folate values, the difference in mean folate levels between the experimental and control groups at 6 months was not significant (*β* = 0.48, *p* = 0.525), suggesting that the fortified rice intervention did not have a measurable impact on folate status over the study period.

Vitamin B_12_ level among the control group decreased from 431.15 ± 252.84 pg./L to 256 ± 118.07 pg./L, (*p* < 0.001), while in the experimental group, it decreased from 522.44 ± 300.12 pg./L to 226.53 ± 79.39 pg./L (*p* < 0.001). The ANCOVA model was found to be statistically significant, *F*(2, 65) = 26.18, *p* < 0.001, and explained approximately 44.6% of the variance in endline vitamin B_12_ concentrations (Adjusted *R*^2^ = 0.429). Baseline vitamin B_12_ was found to be a significant predictor of 6-month values (*β* = 0.24, *p* < 0.001), indicating that individuals with higher baseline levels tended to maintain higher levels over time. However, after adjusting for baseline values, participants in the experimental group had significantly lower serum vitamin B_12_ concentrations at 6 months compared to the control group (*β* = −51.27, *p* = 0.008). The finding suggested that the fortified rice intervention was associated with a decline in vitamin B_12_ status over the study period.

## 4 Discussion

To our knowledge, this is the first field trial on the impact assessment of fortified rice among the tribal population of Gujarat. In this study, impact of routine consumption of fortified rice along with routine diet was studied. This study showed that consumption of fortified rice along with regular meals helped in improving hemoglobin levels among the experimental group at 6 months (Hb:0.55 g/dL) and at 1 year (Hb:0.52 g/dL); however, improvement in serum ferritin, folate, and vitamin B_12_ levels were not observed at the 6-month interval. One plausible reason for the lack of impact was the relatively low level of iron, folate, and vitamin B_12_ fortification levels in the TPDS rice, which contained 4.2 mg of iron, 7.5 12.5 μg of folate, and 0.075–0.125 μg of vitamin B_12_ per 100 grams of fortified rice. This is notably lower than the additional iron levels used in several previous studies that demonstrated positive outcomes.

Literature review revealed that the study carried out by Thankachan et al. (20) used additional iron levels of 6.5 mg/100 g (low iron group) and 12.5 mg/100 g (high iron group); Radhika et al. (21) used 18 mg per 125 g of fortified rice; Sarkar et al. (22) employed sodium iron EDTA (Na₂FeEDTA) at 35–40 ppm; Zimmermann et al. (23) used 13.4 mg iron per day; Beinner et al. (24) used 10.4 mg/100 g; Hotz et al. (25) used 26.6 mg/100 g; Pinkaew et al. (26) used 20 mg/100 g; Ara et al. (27) used 6 mg/100 g; Hardinsyah et al. (28) used 9.8 mg/100 g; and Arcanjo et al. (29) used as much as 102 mg/100 g. All these trials showed significant improvement in micronutrient levels. The comparatively low level of iron in TPDS distributed rice led to the present findings.

The majority of the studied subjects were agricultural farmers, laborers, daily wage laborers, school-going/dropout adolescent girls, etc. Subject-wise per day consumption of fortified rice was also analyzed. Fortified rice was consumed by subjects once a day, mostly in the form of rice and pulse-based mixture *(Dal-Bhat)* or in the form of rice and buttermilk *(Kadi and Khichdi)*. Rice was mostly consumed during dinner time. Commonly consumed pulses were pigeon peas (*Tuver dal*), green gram (*Moong dal*), black gram (*Urad*), spilled chick peas (*Channa dal*), and Bengal gram (*Kala Channa*). On average, 70–100 g of raw fortified rice were consumed per day, amounting to approximately 2.1–3 kg per month per subject. According to the Food Safety and Standards Authority of India (FSSAI), each kilogram of fortified rice should contain approximately 28.5–40.2 mg of iron, 75–125 μg of folate, and 0.75–1.25 μg of vitamin B_12._ The average fortified rice kernels (FRK) content in the rice samples was 9.65 g/kg of fortified rice. Based on the National Accreditation Board for Testing and Calibration Laboratories (NABL) lab report, the average iron content in fortified rice samples was 40.13 mg/Kg, folic acid 93.66 μg/Kg, and vitamin B_12_ 0.89 μg/kg, respectively. All the micronutrients were found at the upper level, as prescribed by the FSSAI. After calculating the overall intake of fortified rice, it was found that 12.6–18 Kg of fortified rice was consumed by the subjects in 6 months. On average, approximately 52.92–75.6 mg of additional iron was received from the TPDS procured fortified rice. An average iron intake of 15.4 mg (11.2 + 4.2) was consumed every day by the subjects of the experimental group compared to 11.8 mg in the control group.

According to FSSAI, ferric pyrophosphate or sodium iron EDTA can be used in wheat and rice; however, sodium iron EDTA was not suitable for rice as it causes color changes; therefore, only ferric pyrophosphate was recommended by the FSSAI. Studies on the bioavailability of ferric pyrophosphate indicates a relative bioavailability ranging from 21% to 74% (30, 31), which may limit its efficacy in addressing micronutrient deficiencies. Additionally, seasonal variation appears to influence nutrient levels. A study carried out by (32) showed that iron and protein intake were higher during the wet season (5.7 mg and 16.9 g) compared to the dry season (4.5 mg and 15.9 g), respectively, among children. Similarly, the mean hemoglobin level was higher in the wet season (11.4 g/dL) compared to the dry season (10.6 g/dL) at *p* = 0.001. According to Valencia-Vera et al. (33), solar radiation causes photodegradation of folate *in vitro* and in human skin. Both the average levels and the percentage of individuals with vitamin B_9_ levels below 3.0 ng/mL were lower in winter. The risk of developing folate deficiency was 1.37 times higher in the summer season compared to the winter season. Seasonal transitions, particularly from winter to summer, significantly reduced the levels of folate and vitamin B_12_. According to Wickham et al. (34), during March to June, total dietary folate intake among nulliparous Dublin women was 114 μg per day, while from November to February, it was found to be 158 μg/day. Mean serum folate level during summer months (March to June) was 3.6 μg/L, while it increased to 5.6 μg/L during the winter months (November to February).

A similar pattern was also observed in the present study. Baseline blood samples were collected in October, while endline samples were collected in April. The observed fluctuations in ferritin, folate, and vitamin B_12_ levels were attributed to seasonal factors. Additionally, in regions such as Gujarat, where very high temperatures (41°C–49 °C) prevail in the summer seasons (35), nutrient intake tends to decline during the summer compared to the winter season. In the winter season, locally available green leafy vegetables, such as spinach, fenugreek, dill leaves *(Saag)*, and millets, particularly pearl millet *(Bajri no rotlo)*, was consumed on a regular basis. Comparing the nutrient intake, it was also observed that the availability and consumption of a diverse range of fruits and vegetables were found to be more in the winter season compared to the summer season. In addition to that, the consumption of tea and coffee was an integral part of the local diet. All the respondents found to consume approximately three to four cups of tea and one to two cups of coffee in a day (with breakfast, before lunch, and in the evening time). Studies show that consumption of tea/coffee has a profound impact on iron absorption as it contains monomeric flavanols, polyphenols, and chlorogenic acid that form non-absorbable iron complexes, which leads to poor iron absorption (36). All these factors, such as use of ferric pyrophosphate, dosages of fortificants, consumption of tea/coffee, climatic factors, seasonal changes in dietary pattern, availability of diverse food groups, dietary combinations, storage conditions of micronutrients, particularly at food corporation godowns significantly reduced micronutrient levels.

## 5 Conclusion

India’s strategy for addressing micronutrient deficiencies largely centers on the distribution of food grains. It is equally essential to give importance to non-nutritional factors. Studies on anemia indicate that it is a complex, multifactorial health issue; while iron deficiency remains the primary contributor, regional diversity in dietary habits also adds further complexity. In such a context, nutrition interventions like food fortification must be approached with caution. Although fortificants are often added in higher concentrations, the storage conditions of food grains—particularly at Food Corporation of India warehouses and Fair Price Shops—need to be closely monitored. This is especially important for heat- and light-sensitive nutrients, such as folate, which can degrade under poor storage conditions. Some improper practices, such as selling fortified rice in the open market, should be closely monitored. There is a need to reform the ration card system so that only the underprivileged beneficiaries can benefit from the scheme. Additionally, there was a limited awareness about sickle cell and Thalassemia, deworming, iron and folic acid supplementation, hygiene, and sanitation etc. These issues must also be prioritized alongside food-based interventions to effectively combat anemia. It is recommended that dedicated, grassroots-level counseling and public education campaigns should be conducted on a priority basis. These efforts can help mitigate incidences of intended misuse of fortified foods, thereby increasing acceptance and efficacy of the program.

## Data Availability

The original contributions presented in the study are included in the article/Supplementary material, and further inquiries can be directed to the corresponding author.
