# Supplementary material for: Impact assessment of fortified rice under the public distribution system: evidence from the tribal regions of Gujarat
Source: Front Public Health. 2026 Apr 1;14:1757408. doi: 10.3389/fpubh.2026.1757408 (PMC13079153; doi:10.3389/fpubh.2026.1757408)
Supplement: Supplementary file 1 [file Table_1.docx]

**SURVEY SCHEDULE-**

| **Date:** | | | | | **Subject code:** | | | | |
| --- | --- | --- | --- | --- | --- | --- | --- | --- | --- |
| 1. | | Name of the Taluka |  | | | | | | |
| 2. | | Name of the Village |  | | | | | | |
| 3. | | Type of ration card | APL/BPL/AAY | | | | | | |
| 4. | | Name of the Individual |  | | | | | | |
| 5. | | Age |  | | | | | | |
| 6. | | Gender | Female | Male | | | | | |
| 7. | | Religion 1. Hindu 2. Muslim 3. Jain 4. Christian 5. Any other | | | | | | | |
| 8. | | Type of Diet 1. Vegetarian 2. Non-Vegetarian | | | | | | | |
| 9. | | Contact No. |  | | | | | | |
| 10. | | Weight |  | | | | | | |
| 11. | | Height |  | | | | | | |
| 12. | | BMI |  | | | | | | |
| 13. | | MUAC |  | | | | | | |
| 14. | | Haemoglobin |  | | | | | | |
| **KUPPUSWAMMY SOCIOECONOMIC SCALE (Version, 2021)** | | | | | | | | | |
| 1 | Name of the household he | |  | | | Age |  | Gender |  |
| 2 | Occupation of the household head | | \| - 1. Legislators, senior officials & Managers \| \| --- \| \| - 1. Professionals \| \| - 1. Technicians & associate professionals \| \| - 1. Clerks \| \| - 1. Skilled workers and shop & market sales workers \| \| - 1. Skilled agricultural & fishery workers \| \| - 1. Craft & related trade workers \| \| - 1. Plant and Machine Operators & Assemblers \| \| - 1. Elementary occupation \| \| - 1. Unemployed \| | | | | | | |
| 3 | Education of the Head of the Family | | \| 1. Profession or Honors \| \| --- \| \| 1. Graduate \| \| 1. Intermediate or diploma \| \| 1. High school certificate \| \| 1. Middle school certificate \| \| 1. Primary school certificate \| \| 1. Illiterate \| | | | | | | |
| 4 | Family type | | 1. Nuclear  2. Joint  3. Extended | | | | | | |
| 5 | Number of family Members | | \|  \| **Male** \| **Female** \| **Total** \| \| --- \| --- \| --- \| --- \| \| Adult \|  \|  \|  \| \| Children \|  \|  \|  \| | | | | | | |
| 6 | Education of the respondent | | 1. Primary  2. Secondary  3. Higher secondary  4. Graduate  5. Illiterate  6. Other | | | | | | |
| 7 | Occupation of the respondent | | 1. Casual  2. Daily  3. Salaried  4. Self-Employment  5. Unemployed  6. Housewife | | | | | | |
| 8 | Average family income (in ₹) | | 1. ≥123,322 2. 61,663-123,321 3. 46129-61,662 4. 30,831-46,128 5. 18,497-30,830 6. 6175-18,496 7. <6174 | | | | | | |
| 9 | Source of drinking water | | - 1. Tap Water (b) Hand pump (c) Tubewell | | | | | | |
| 10 | Functional Toilet | | Yes/No | | | | | | |
| 11 | Type of house | | Kacha/Semipakka/Pakka | | | | | | |
|  | Do you have animals? Yes/NO | | | | | | | | |
|  | How many domestic animals do you have?  (a)1 (b) 2 (c) 3 (d) 4 (e) 5 (f) 6 (g) more than 6 | | | | | | | | |
|  | Which animals/poultry do you rear?   1. Cow (b) Buffalo (c) Goat (d) Poultry birds | | | | | | | | |
|  | Do you have land holding? Yes/No | | | | | | | | |
|  | If Yes, how much land do you have? | | | | | | | | |

**24-hour Dietary Recall**

| **Time** | **Name of the dish** | **Ingredients** | **Quantity** |
| --- | --- | --- | --- |
| Day-1 |  |  |  |
| Breakfast |  |  |  |
| Lunch |  |  |  |
| Dinner |  |  |  |
| Day-2 |  |  |  |
| Breakfast |  |  |  |
| Lunch |  |  |  |
| Dinner |  |  |  |
| Day-3 |  |  |  |
| Breakfast |  |  |  |
| Lunch |  |  |  |
| Dinner |  |  |  |

**CONSENT FORM (ENGLISH)**

The proposed research title is “Effectiveness Evaluation of Fortified Rice and Fortified Wheat Distributed through the Public Distribution System among Tribal Households.

Nutritional anaemia is a major public health concern affecting people of different age groups. The present study is an initiative to assess the role of fortified rice in the prevention of nutritional anemia. Food fortification is a strategy that has received global recognition for its long-term nutritional benefits and acceptability.

The objective of the research is to assess the impact of fortified rice consumption on the general health of men and women aged 15–45 years residing in your locality. The assessment will be carried out using anthropometric measurements such as height, weight, MUAC, 24-hour dietary recall and food frequency, and biochemical parameters (blood samples).

The study involves evaluation at baseline and at the end of the intervention to measure improvement in health status. All dietary, anthropometric, and biochemical data collected will be kept strictly confidential and will be used only for research purposes.

I shall be grateful for your kind cooperation.

**Certificate**

I, ........................................, a resident of village ........................................ of………………………taluka/Block of ……………., voluntarily and with full understanding, give my consent to participate in this research study. I have understood the objectives of the study, and I am aware that I may withdraw from the study at any stage without giving any reason.

If you have any further questions regarding this study, you may contact.

Contact Number ------------------
